# Revealing sound-induced motion patterns in fish hearing structures in 4D: a standing wave tube-like setup designed for high-resolution time-resolved tomography

**DOI:** 10.1242/jeb.243614

**Published:** 2022-01-04

**Authors:** Isabelle P. Maiditsch, Friedrich Ladich, Martin Heß, Christian M. Schlepütz, Tanja Schulz-Mirbach

**Affiliations:** 1 University of Vienna, Department of Behavioral and Cognitive Biology, 1030 Vienna, Austria; 2 Ludwig-Maximilians-University Munich (LMU), Department Biology II, Planegg-Martinsried, 82152 Germany; 3 Swiss Light Source (SLS), Paul Scherrer Institute (PSI), 5232 Villigen, Switzerland

**Keywords:** Auditory structures, Synchrotron radiation, Swim bladder, Retrospective gating, Otolith, Otophysa

## Abstract

Modern bony fishes possess a high morphological diversity in their auditory structures and auditory capabilities. Yet, how auditory structures such as the otoliths in the inner ears and the swim bladder work together remains elusive. Gathering experimental evidence on the *in situ* motion of fish auditory structures while avoiding artifacts caused by surgical exposure of the structures has been challenging for decades. Synchrotron radiation-based tomography with high spatio-temporal resolution allows the study of morphofunctional issues non-invasively in an unprecedented way. We therefore aimed to develop an approach that characterizes the moving structures in 4D (=three spatial dimensions+time). We designed a miniature standing wave tube-like setup to meet both the requirements of tomography and those of tank acoustics. With this new setup, we successfully visualized the motion of isolated otoliths and the auditory structures in zebrafish (*Danio rerio*) and glass catfish (*Kryptopterus vitreolus*).

## Abstract (precis)

**Summary:** To characterize the sound-induced motion of fish auditory structures in 4D, we developed a tomography-compatible standing wave tube-like setup and thereby demonstrated the previously hypothesized rotational motion of otophysan sagittae.

## INTRODUCTION

Modern bony fishes (teleosts) cover a wide range of auditory capabilities (e.g. in terms of auditory sensitivity and the range of detectable frequencies) alongside a great diversity in their auditory structures (Braun and Grande, 2008; Ladich and Fay, 2013; Ladich and Schulz-Mirbach, 2016). Primarily, auditory structures refer to the inner ears including the mineralized otoliths, and can further include the swim bladder or any other gas-filled bladder, especially if these bladders approach or contact the inner ears (i.e. otophysic connection; Braun and Grande, 2008) as found in otophysan fishes. Otophysans such as zebrafish, *Danio rerio* (Cyprinidae), are important model organisms to study deafness and balance disorders in human medicine (e.g. Abbas and Whitfield, 2010). However, identifying the factors determining otolith motion and inner ear stimulation is still challenging in species such as zebrafish that have evolved specialized ancillary auditory structures (Weberian apparatus) for hearing enhancement (e.g. Schulz-Mirbach et al., 2019). Hence, knowledge of the interaction of fish auditory structures remains elusive (Popper et al., 2005; Ladich and Schulz-Mirbach, 2016).

The most straightforward and reliable way to study fish auditory structures ‘in action’ is the direct observation of the sound-induced *in situ* motion of the structures in the test subject. However, the technical challenge of gaining access to these internal structures, moving at amplitudes in the range of a few micrometers and at typical sound frequencies, without altering their response as a consequence of surgical procedures has hampered such research for many years. Only a limited number of experimental studies have investigated the sound-induced motion of the saccular otoliths (de Vries, 1950; Sand and Michelsen, 1978), the swim bladder walls (Popper, 1974; Clarke et al., 1975) or the whole set of auditory structures (Cox and Rogers, 1987). Nowadays, synchrotron radiation-based techniques provide powerful approaches to perform imaging of internal structures at high spatio-temporal resolution non-invasively (Mokso et al., 2015; Rack et al., 2010; Walker et al., 2014). Recent studies using hard X-ray phase contrast imaging at the European Synchrotron Radiation Facility (ESRF, Grenoble, France) provided first insights into the sound-induced motion of fish auditory structures (Schulz-Mirbach et al., 2018, 2020). In these radiographic experiments using a standing wave tube-like tank, the motion of the swim bladder and the otoliths in the inner ears of goldfish (*Carassius auratus*, Otophysa) and the cichlid *Etroplus canarensis* was successfully visualized and motion patterns could be described qualitatively. However, the motion of the structures of interest could only be observed in 2D, i.e. in a certain orientation. In addition, ‘overlying’ structures such as gills or cranial bones, in part, hampered an undisturbed view of the moving auditory structures. Hence, any in-depth insights into the basic principles of the interaction of the auditory structures would require the visualization and characterization of the motion patterns during sound presentation covering the structures' full 3D aspect. A study on the flight motor motion of a blowfly performed at the TOMCAT beamline at the Swiss Light Source (SLS, Villigen, Switzerland) (Mokso et al., 2015; Walker et al., 2014) demonstrated that periodic motion patterns can be evaluated using tomography with high spatio-temporal resolution.

In mammals and birds, the inner ears show a rather clear division of labor consisting of a vestibular part (semicircular canals and in a wider sense the utricle and the saccule) and the portion serving the auditory sense, i.e. the lagena or cochlea (Manley and Clack, 2004; Witmer et al., 2008; Ladich, 2019). In these terrestrial vertebrates, sound pressure is the primary stimulus (Ladich, 2019; Fay and Popper, 2005). In contrast, in teleosts (and fishes in general), the otolith end organs, namely utricle, saccule and lagena, seem to serve both senses (‘mixed function hypothesis’; Platt and Popper (1981). Moreover, fish inner ears detect sound through particle motion (Rogers et al., 1988). The ability to additionally make use of the pressure component of sound is developed in several teleost groups (e.g. Otophysa, Clupeiformes, Mormyridae, Anabantiformes, several sciaenid species; Braun and Grande, 2008; Hawkins, 1993; Ramcharitar et al., 2006; Horodysky et al., 2008). Sound pressure detection in fishes is possible when the swim bladder or any gas-filled bladder acts as a pressure-to-displacement transducer (Rogers et al., 1988). This is most effective if the bladder is connected to the inner ears through anterior extensions or mechanically coupled by a chain of ossicles and ligaments as in otophysan fishes (Rogers et al., 1988; Schulz-Mirbach et al., 2019). Sound-induced pressure fluctuations provoke the compression and decompression of the gas in the bladder and result in the oscillation of the swim bladder walls. The oscillating walls then function as a secondary sound source, creating local particle motion, ultimately setting the otoliths in motion (Rogers et al., 1988; Schulz-Mirbach et al., 2019). As both sound components, acoustic particle motion and sound pressure, may play a role in stimulating fish inner ears, we set out to adapt the standing wave tube-like setup used at the ESRF for tomography at the SLS. The setup at the ESRF consisted of a horizontal 2L Plexiglas^®^ tube equipped with a miniature inertial shaker at each end which could be driven under different phase conditions to create either a sound pressure or particle motion maximum in the tube center. The intended setup adaption implied, among others, a miniaturization from a horizontal 2 l tank to an upright 14.1 ml (one-shaker setup) or 40.8 ml (two-shaker setup) tube, respectively.

In our study, we aimed to develop a setup that enables non-invasive experiments characterizing the 3D motion of fish auditory structures during sound presentation, such that these experiments can be performed under sound-induced particle motion or sound pressure conditions. In the following, we characterize the approach, focusing on the acoustic setup.

## MATERIALS AND METHODS

### Samples and study organisms

Initial tests to optimize the setup parameters such as sound amplitude and frequency, exposure time, X-ray energy and number of images per scan were performed with an isolated left saccular otolith from a goldfish, *Carassius auratus* (L. 1758) [otolith ex coll. T.S.-M., standard length (SL) 49 mm; tested in the one-shaker setup]. Subsequently, the *in situ* motion patterns of the intact auditory system were investigated in freshly euthanized specimens of zebrafish *Danio rerio* (Hamilton 1822) (*N*=3, SL 22 mm; tested in the one-shaker setup) and glass catfish *Kryptopterus vitreolus* Ng and Kottelat 2013 (*N*=2, SL 46 mm and 56 mm, respectively; tested in the two-shaker setup). We chose zebrafish and glass catfish because of their small size and the elongate/cylindrical body shape ideally suited for the dimensions of the small test tube. The three studied zebrafish specimens of the wild-type line ‘AB’ (provided by Prof. Anna Jazwinska Müller, University of Fribourg, Switzerland) originated from one hatching (25 October 2018). The two glass catfish individuals were obtained from a local aquarium trader (Aquarium & Teich AG, Villmergen, Switzerland). Prior to the experiments, fish were kept for 1 week in 20–30 l tanks equipped with internal filters at a temperature of 23°C and fed once a day with commercial flake food at the TOMCAT beamline. Before each experiment, the test subject was euthanized with a 0.4% solution of tricaine methanesulfonate (Sigma Aldrich) buffered with sodium bicarbonate. The fish was introduced into the X-ray beam when opercular movements had completely ceased and no vestibulo-ocular reflex was discernible. The experiments were approved by the cantonal veterinary service Aargau, Switzerland (approval no. 75725 and 75734). All experiments at the ESRF were conducted in accordance with ethical guidelines, i.e. article 3, point 1 of the EU directive 2010/63, accepted by all EU countries. As no living animals were used during the imaging procedure, no ethical approval was required.

### Setup design

**Fig. 1.**
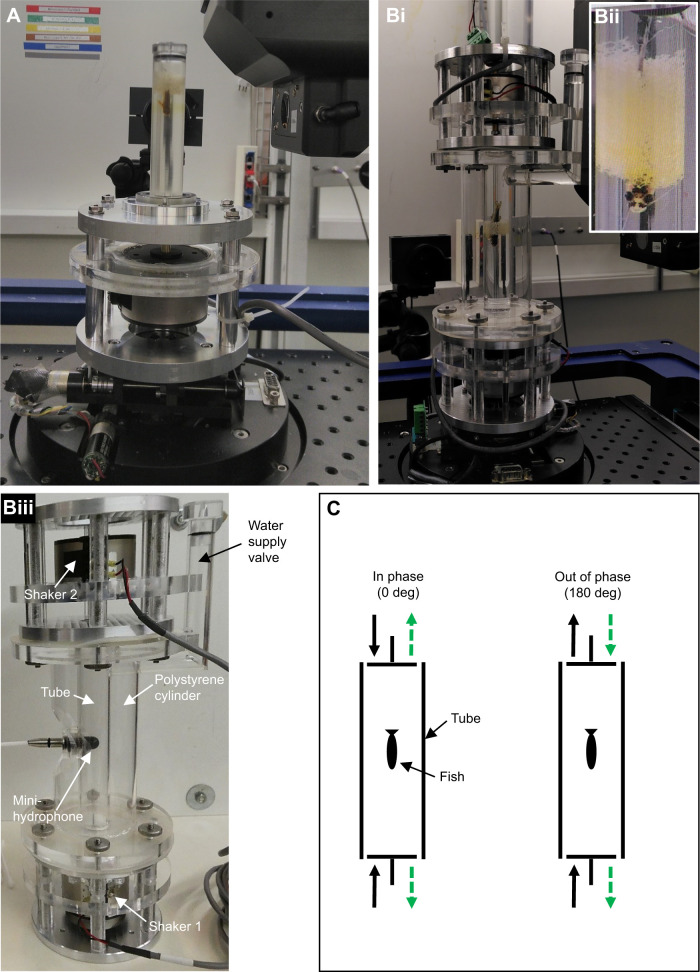
**Overview of the one-shaker and two-shaker setup.** (A) One-shaker setup (14.1 ml tube), in which the shaker is mounted at the bottom beneath the tube. (Bi) Two-shaker setup (40.8 ml tube), in which a second shaker is mounted on top of the tube. (Bii) The test subject is ‘fixed’ in the center of the tube using a piece of porous foam wrapped around the fish. (Biii) Modification of Bi which allows insertion of the miniature hydrophone in the center of the tube to measure sound pressure level (SPL). (C) Illustration of how an ideal standing wave tube working under the 0 in-phase condition results in maximum sound pressure in the center of the tank whereas driving the shakers 180 deg out of phase creates maximum particle motion in the tank center.

To meet the requirements of tomographic imaging, namely the need to illuminate the sample with X-rays from all directions in the imaging plane and to be able to rotate it by at least 180 deg around a direction perpendicular to the X-ray beam, we used small transparent test tubes to minimize X-ray absorption by the tube walls and the water body in the tube. First, we conducted experiments (March and June 2019) with a one-shaker setup consisting of a transparent (Plexiglas^®^) tube and one miniature inertial shaker (2002E, PCB Synotec) mounted at the bottom of the tube. The test tube had an inner diameter of 15 mm, a height of 80 mm and a wall thickness of 2 mm, resulting in a volume of 14.1 ml (Fig. 1A). Subsequently (September 2020), we upgraded the system by adding a second miniature inertial shaker (2002E, PCB Synotec) on top of a longer test tube. In this two-shaker setup (i.e. standing wave tube-like setup; Fig. 1B), the test tube had an inner diameter of 19 mm, a height of 144 mm, a wall thickness of 2 mm with a larger volume of 40.8 ml. To stabilize the upper shaker and the whole setup, the system was equipped with an outer cylinder which had an inner diameter of 53 mm, a height of 142 mm and a wall thickness of 3 mm.

Pure tone stimuli were transferred to the (degassed) water body by the (one or two) miniature inertial shaker(s) driving a short metal rod (a) fitting into the lid of a 1.5 ml Eppendorf tube that was glued to the center of a rubber membrane which sealed the bottom of the tube (one-shaker setup, Fig. 1A) or (b) with each shaker transmitting the vibration to the water body through a knurl-head screw (diameter: 16 mm, thickness: 3 mm) attached to a silicon membrane (thickness: 1 mm; two-shaker setup, Fig. 1Bi–iii). The two-shaker setup was run as a standing wave tube-like setup with the two shakers either driven 0 deg in phase or 180 deg out of phase. In an ideal standing wave tube (Fig. 1C), the condition ‘0 deg in phase’ results in maximum sound pressure in the center of the tube while there is no particle motion present. Accordingly, the 180 deg out of phase condition creates maximum particle motion in the center of the tube while there is no sound pressure (Hawkins and MacLennan, 1976).

The samples were held in place in the center of the test tube using a piece of porous foam (thickness: 10 mm) while fixing the sample inside the foam at only a few points to allow for a ‘free’ motion (Fig. 1Bii). Before fixing the samples, air bubbles were removed from the foam by squeezing it underwater.

### Sound stimuli and characterization of the sound field

In previous studies (Schulz-Mirbach et al., 2018, 2020), a stimulus frequency of 200 Hz resulted in a successful observation of the 2D *in situ* motion of otoliths applying hard X-ray phase contrast imaging. Accordingly, we tested stimuli of 100 and 200 Hz (one-shaker setup). To optimize the setup to higher frequencies more relevant in the context of the swim bladder–inner ear interaction (Myrberg and Spires, 1980), we additionally tested stimuli of 350 and 450 Hz (two-shaker setup). We used two different amplifier settings to take into account potential effects of different sound (pressure) levels on the motion of the auditory structures (Table S1).

The stimuli were generated by the software CoolEdit 2000 (Syntrillium Software Corp., Phoenix, AZ, USA) as ‘simple’ pure tones in the case of the stimuli tested in the one-shaker system. In the case of the two-shaker setup, the stimuli were generated as 0 and 180 deg phase-shifted pure tones per test frequency. Stimuli were presented using the internal sound card (Conexant 20561 SmartAudio HD and NVIDIA High-Definition Audio) of a laptop (Lenovo ThinkPad T500 and Lenovo ThinkPad P53) which was connected to the shaker(s) through an amplifier (S.M.S.L. SA 36A Pro, Shenzhen ShuangMuSanLin Electronic Co., Ltd, Shenzhen, China).

Acoustic measurements of the ambient noise in the hutch of the beamline and of the test frequencies were performed before or after the actual experiments. Simultaneous acoustic measurements were not possible because either the miniature hydrophone (Brüel & Kjær 8103, sensitivity: −211 dB re. 1 V µPa^−1^; diameter: 9.5 mm) would have been affected by the X-ray beam or the sound pressure level (SPL) would have been evaluated in places other than the location of the sample, i.e. other than in the center of the tube. SPL was measured using the miniature hydrophone (see Fig. 1Biii) connected to a sound level meter (Brüel & Kjær 2238 Mediator) which had been calibrated with a hydrophone calibrator (Brüel & Kjær 4229) beforehand.

The ambient noise in the center of the tube was recorded using the software CoolEdit 2000 and the miniature hydrophone connected to the internal sound card of the laptop via an amplifier (36B2, constructed at the University of Vienna; including an electrical grounding). The ambient noise spectrum was then analyzed using the sound analyzing software STX 3.7.8 (Acoustics Research Institute, Austrian Academy of Sciences, Vienna, Austria). Tone stimuli in the test tube were recorded using CoolEdit 2000 with the miniature hydrophone and the 36B2-amplifier connected to the laptop while playing the tone stimuli via the same laptop using the shaker(s) and the S.M.S.L. SA 36A Pro amplifier.

### Synchrotron X-ray tomography

At the TOMCAT beamline of the SLS, X-rays are produced by a 2.9 T superbending magnet located 25 m from the sample. The full white beam spectrum of the source was filtered to reduce the incoming X-ray intensity. A Ce-doped LuAG scintillator (thickness: 150 µm), transforming the X-ray image to visible light, was placed at distances of 300, 200, 120 and 70 mm behind the sample to test different levels of edge enhancement from the soft tissues. Finally, a scintillator distance of 70 mm was chosen to optimize the phase contrast of the radiographs. The resulting edge-enhanced visible light image of the sample on the scintillator was then magnified using a custom-made, high numerical-aperture microscope (Optique Peter, Lentilly, France) with 4-fold magnification and recorded with a GigaFRoST 12-bit detector system (Mokso et al., 2017) with a native pixel size of 11 µm, resulting in an effective pixel size of 2.75 µm. Full scans of the oscillating samples (tomography with sound, i.e. 4D stacks) were acquired at frame rates of around 2 and 5 kHz while the sample was rotated over 180 deg. Static scans of the same samples (tomography without sound, i.e. 3D stacks) were recorded with 1500 projections over 180 deg at exposure times of 1.0–1.5 ms. The experimental parameters for all dynamic samples are detailed in Table 1.

**Table 1.**
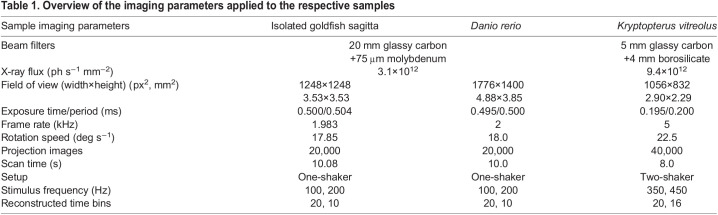
Overview of the imaging parameters applied to the respective samples

Projection-guided retrospective gating and tomographic reconstruction was performed based on the approach described by Mokso et al. (2015). Hence, for each stimulus frequency, projections taken from different angles but at the same phase during the stimulus presentation were grouped into respective time bins. This resulted in 20 time bins for the 100 Hz and 350 Hz stimuli, 10 time bins for the 200 Hz stimulus, and 16 time bins for the 450 Hz stimulus. For each time bin, a single distance propagation-based phase retrieval (δ/β=50) based on Paganin's algorithm (Paganin et al., 2002) was applied to the projections from which the 3D volume datasets were reconstructed using the gridrec reconstruction algorithm (Marone and Stampanoni, 2012).

### Segmentation and generating the 3D surfaces

To qualitatively evaluate the motion patterns under different setup conditions, we segmented and labeled the 3D volume of the saccular otoliths in the reconstructed datasets using Dragonfly v. 2021.1 (Object Research Systems Inc.). We focused on the otophysan saccular otoliths for two reasons. First, this structure showed a rotational movement induced by sound pressure in a previous study which could be easily identified even in radiographs (Schulz-Mirbach et al., 2020) and was also in line with the motion pattern hypothesized by experts in the last century (von Frisch, 1938; de Burlet, 1929; Wohlfahrt, 1932). Second, the 0 deg in phase and 180 deg out of phase conditions in the previous study clearly differed from each other by the evoked motion pattern of this otolith, with the rotational motion only identifiable under the 0 deg in-phase condition.

The image stacks were cropped along the *x*-, *y*- and *z*-axes to reduce stack size without performing resampling, i.e. the isotropic voxel size of 2.75 µm was retained for all steps. We labeled the otoliths in every third (200 Hz, 450 Hz) or every fifth (100 Hz, 350 Hz) timing bin of each 4D stack applying the same threshold-based segmentation settings (method: upper or lower Otsu). Any manual corrections of the segmented structures were kept to a minimum.

For the isolated goldfish sagitta embedded in foam, a threshold-based approach did not yield a satisfying result. We therefore trained a deep-learning U-net model in Dragonfly v. 2020.1 (type: semantic segmentation using a mask, patch size: 80, stride ratio: 1, batch size: 8, epochs: 14, loss function: categorical cross-entropy, data augmentation set to 3×) which resulted in a more accurate and reproducible labeling of the sagitta than using any threshold-based segmentation tool. However, some manual corrections were necessary, especially at the ‘free’ ventrolateral wing (see fig. 4 of von Frisch, 1938) of the sagitta. To evaluate the success and to estimate the accuracy of the segmentation process, volume and surface area of the labeled structures across the studied timing bins were compared with the values obtained from the respective structure visualized without sound exposure.

**Fig. 2.**
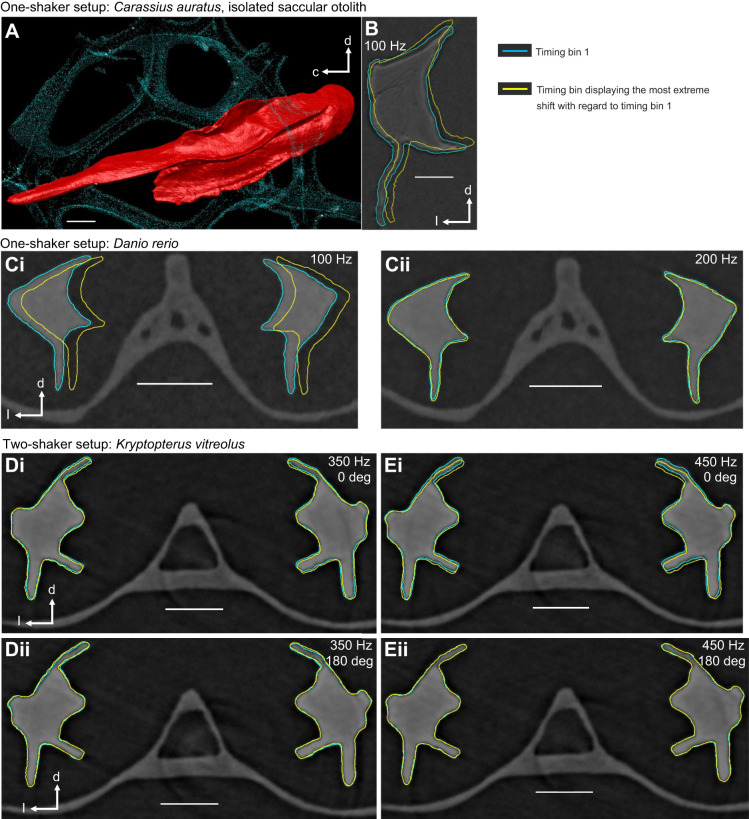
**Motion patterns of the saccular otoliths studied in the one-shaker and two-shaker setup.** (A) 3D reconstruction of the isolated goldfish sagitta (medial view, red) ‘embedded’ in the piece of foam (turquoise). The dashed line indicates the location of the transverse section shown in B. (B) The overlay of the contours shown for two timing bins reveals a slight rotational motion. c, caudal; d, dorsal; l, lateral. (Ci) Sagittae in zebrafish (standard length, SL 22 mm) subjected to a 100 Hz stimulus in the one-shaker setup display a distinct translational but no rotational movement. (Cii) A faint rotational motion of the sagittae was visible when the zebrafish was subjected to the 200 Hz stimulus. (D,E) A clear rotational motion of the sagittae in *Kryptopterus vitreolus* (SL 46 mm) was observed when the fish was subjected to 350 Hz (Di) or 450 Hz (Ei) driving the two shakers 0 deg in phase. This rotational movement was faint (Dii) or absent (Eii) when the shakers were driven 180 deg out of phase. Scale bars: 100 µm.

To produce the overlay of the sagitta outlines shown in Fig. 2, one sub-stack within the 4D stack was rotated applying the ‘translate/rotate tool’ until both sagittae were displayed in an exact transverse section on the *x*–*y*-plane. The transformation was then applied to the labels of the other sub-stacks. Subsequently, surfaces (‘meshes’) were generated and smoothed (number of iterations: 1) without using the resampling option.

## RESULTS AND DISCUSSION

### Setup acoustics

In the two setups, the ambient noise level (one-shaker setup: 112.4, 115.7 dB; two-shaker setup:123.4 dB) was 33.6–50.6 dB (one-shaker setup) and 42.8–64.3 dB (two-shaker setup) below the SPL measured during the presentation of any of the stimulus frequencies (148.0–187.7 dB re. 1 µPa; Table S1). In the one-shaker setup, the spectrum of the recorded 100 Hz stimulus revealed peaks at 100 Hz followed by harmonics of this frequency with higher levels (Fig. S1A). In contrast, the spectrum of the recorded 200 Hz stimulus displayed a distinct peak at the stimulus frequency and harmonics at much lower levels (Fig. S1B). In the two-shaker setup, the spectra of the recorded 350 Hz (Fig. S1C_1_,C_2_) and the 450 Hz (Fig. S1D_1_,D_2_,E_1_,E_2_) stimuli showed clear peaks at the stimulus frequencies for both phase conditions, i.e. the 0 deg in-phase and 180 deg out-of-phase mode. However, the spectrum of the recorded 450 Hz stimulus, especially under the 0 deg in-phase condition and amplifier setting ‘b’ (SPL 187.7 dB re. 1 µPa), also revealed high harmonics of this frequency (see the peak at 900 Hz in Fig. S1E_1_). In addition, the applied phase conditions differed by at least 10 dB (Table S1) with the 0 deg in-phase condition displaying higher SPLs than the 180 deg out-of-phase condition at the same stimulus frequency and using the same amplifier setting.

### Sound-induced motion of the sagittae

#### One-shaker setup

The isolated goldfish sagitta embedded in porous foam (Fig. 2A) showed a rotational motion (Fig. 2B) when subjected to the 100 Hz stimulus. In zebrafish, the sagittae revealed different *in situ* motion patterns depending on the test frequency. A simple medio-lateral shift of the two sagittae in the same direction (Fig. 2Ci) was observed when the fish were subjected to the 100 Hz stimulus. In contrast, for the 200 Hz stimulus, the zebrafish sagittae displayed a weak left–right symmetrical rotational movement (Fig. 2Cii).

#### Two-shaker setup

The *in situ* motion patterns of the sagittae in the glass catfish were the same for the two test frequencies and just differed with regard to the phase condition in which the two shakers operated. When the shakers were driven 0 deg in phase, both sagittae displayed a rotational motion (Fig. 2Di,Ei; Movie 1). When the shakers were driven 180 deg out of phase, the sagittae showed only a weak (Fig. 2Dii) or no (Fig. 2Eii) rotational motion.

In our study, we aimed to miniaturize the standing wave tube-like setup used previously for 2D radiography plus time measurements at the ESRF to adapt it for high-resolution tomography (4D=three spatial dimensions + time) at the SLS. Our developed miniature standing wave tube-like setup proved to be effective in characterizing the motion patterns of fish auditory structures covering the full three-dimensionality of their morphology. In the following, we will briefly discuss the observed motion patterns with respect to both setups used in this study as well as the potential for further improvements of the miniature standing wave tube-like setup.

### Relationship between setup conditions and observed motion patterns

The rotational motion of the isolated sagitta in the one-shaker setup and of the glass catfish sagittae *in situ* in the two-shaker setup indicates that the water-soaked porous foam fixing the sample (sagitta or fish) provided enough degrees of freedom to allow the sample to move ‘freely’. This means that the isolated otolith or the otoliths *in situ* do not just move along the main axis of sound propagation (i.e. along the tube axis). For the isolated goldfish sagitta, the points at which the foam holds the sample in place directly affect the motion pattern of the otolith in the sound field. Besides the foam limiting the movement of the whole fish, the native attachment of the otolith to the sensory epithelium through the otolithic membrane and the surrounding membrane of the end organ primarily limit the degrees of freedom for the otolith to move *in situ* (Dunkelberger et al., 1980; Platt and Popper, 1981).

In the case of the two-shaker setup, the distinct rotational motion of the glass catfish sagittae is suggestive of a quite effective separation of sound pressure and sound-induced particle motion in the tube at the tested frequencies (350 Hz, 450 Hz). This rotational motion of the sagittae was expected based on our previous study in goldfish at the ESRF using a standing wave tube-like tank (Schulz-Mirbach et al., 2020) and according to former theoretical considerations (von Frisch, 1938; Freiin von Boutteville, 1935; de Burlet, 1929). In our study on goldfish, the 0 deg in-phase condition and thus the sound pressure maximum in the center of the tank, induced the oscillation of the swim bladder walls which, in turn, resulted in the motion of the coupled Weberian ossicles and finally in a rotation of the saccular otoliths. The motion of the Weberian ossicles was hypothesized to provoke a fluid flow in the sinus impar and the canalis transversus communicans connecting the two saccules to each other. The incoming fluid from the canalis transversus communicans then should impinge on the ‘free’ wing of the sagitta, inducing a rotational motion (von Frisch, 1938; Freiin von Boutteville, 1935; de Burlet, 1929). Accordingly, the rotational sagitta motion we observed in our miniature standing wave tube-like setup when subjecting the fish to the 0 deg in-phase condition perfectly matches this prediction.

The rather high SPLs (>148 dB re. 1 µPa) might influence the motion patterns of the auditory structures. A previous study using 2D radiography tested a series of different SPLs while subjecting fish to a 200 Hz stimulus (Schulz-Mirbach et al., 2020). The amplitudes of the moving saccular otoliths and Weberian ossicles indicated a decrease in motion with decreasing SPL values while the motion patterns themselves were the same regardless of the SPL tested. In future studies, we will conduct similar tests applying descending SPL series to investigate the effects of the moving auditory structures during 4D tomography.

### Setup performance

The small test tubes seem to be less suitable to study the motion of fish auditory structures at low frequencies. In the one-shaker setup, this was indicated by the asymmetrical shift of both zebrafish sagittae at 100 Hz together with the spectrum displaying multiple harmonics of the stimulus frequency. In addition, we observed that SPLs measured for the 100 and 200 Hz stimuli in the two-shaker setup did not meet the expected conditions for the two conduction modes (0 deg versus 180 deg) in a standing wave tube. For these frequencies, the SPLs were similar or even slightly larger under the 180 deg out-of-phase mode compared with the 0 deg in-phase mode (data not shown). These observations are in line with theoretical considerations on the correlation between wave length and distance of sound propagation (Rogers and Cox, 1988) and former experiments on fish hearing using standing wave tubes (Myrberg and Spires, 1980; Hawkins and MacLennan, 1976). The 100 Hz and 200 Hz stimuli have long wavelengths of 14.8 m and 7.4 m compared with the dimensions of our test tubes. This is why former studies conducting experiments in standing wave tube setups used long test tubes when subjecting fishes to low frequency stimuli (Myrberg and Spires, 1980: 5 m, tested frequencies 100–800 Hz; Hawkins and MacLennan, 1976: 0.8 m, tested frequencies 10–250 Hz). We are also aware that the hydrophone itself might affect the acoustics of the test tube. To our knowledge, the miniature hydrophone used is, however, currently the smallest available device to measure underwater SPLs. Notwithstanding these issues, our two-shaker setup allowed for a sufficient separation of sound pressure and sound-induced particle motion at 350 Hz and 450 Hz, which is underlined by the differences in sagitta motion due to the 0 deg in-phase and 180 deg out-of-phase condition, respectively. In a subsequent study, we will further optimize our setup to test stimuli in the kilohertz range and at lower SPLs along with direct measurements of particle motion in the tube.

In summary, our miniature standing wave tube-like setup in combination with the high-resolution time-resolved tomography is an approach capable of capturing the motion patterns of fish auditory structures while also covering the structures' three-dimensionality. With the envisaged optimizations, our setup will be even more powerful and will allow us to test hypotheses on the impact of the morphological variation of fish auditory structures on their motion patterns in a sound field.

## Supplementary Material

Supplementary information

## References

[JEB243614C1] Abbas, L. and Whitfield, T. T. (2010). The zebrafish inner ear. In *Fish Physiology* (ed. S. F. Perry, M. Ekker, A. P. Farrell and C. J. Brauner), pp. 123-171. Academic Press.

[JEB243614C2] Braun, C. B. and Grande, T. (2008). Evolution of peripheral mechanisms for the enhancement of sound reception. In *Fish Bioacoustics* (ed. J. F. Webb, R. R. Fay and A. N. Popper), pp. 99-144. New York: Springer.

[JEB243614C3] Clarke, N. L., Popper, A. N. and Mann, J. A. Jr. (1975). Laser light-scattering investigations of the teleost swimbladder response to acoustic stimuli. *Biophys. J.* 15, 307-318. 10.1016/S0006-3495(75)85821-8 1125389 PMC1334692

[JEB243614C4] Cox, M. and Rogers, P. H. (1987). Automated noninvasive motion measurement of auditory organs in fish using ultrasound. *J. Vibration Acoustics Stress Reliability Design* 109, 55-59. 10.1115/1.3269395

[JEB243614C5] De Burlet, H. M. (1929). Anatomisches zur Hörfähigkeit der Siluroiden. *Anat. Embryol.* 89, 11-27. 10.1007/BF02117946

[JEB243614C6] De Vries, H. (1950). The mechanics of the labyrinth otoliths. *Acta Otolaryngol.* 38, 262-273. 10.3109/00016485009118384 14856657

[JEB243614C7] Dunkelberger, D. G., Dean, J. M. and Watabe, N. (1980). The ultrastructure of the otolithic membrane and otolith in the juveniIe Mummichog, *Fundulus heteroclitus*. *J. Morphol.* 163, 367-377. 10.1002/jmor.1051630309 30184993

[JEB243614C8] Fay, R. R. and Popper, A. N. (2005). Introduction to sound source localization. In *Sound Source Localization*, pp. 1-5. New York: Springer.

[JEB243614C9] Freiin Von Boutteville, K. (1935). Untersuchungen über den Gehörsinn bei Characiniden und Gymnotiden und den Bau ihres Labyrinthes. *Z. Vergl. Physiol.* 22, 162-191. 10.1007/BF00586498

[JEB243614C10] Hawkins, A. D. (1993). Underwater sound and fish behaviour. In *Behaviour of Teleost Fishes* (ed. T. J. Pitcher), pp. 114-151. London: Chapman and Hall.

[JEB243614C11] Hawkins, A. D. and Maclennan, D. N. (1976). An acoustic tank for hearing studies on fish. In *Sound Reception in Fish* (ed. A. Schuijf and A. D. Hawkins), pp. 149-169. Amsterdam: Elsevier.

[JEB243614C12] Horodysky, A. Z., Brill, R. W., Fine, M. L., Musick, J. A. and Latour, R. J. (2008). Acoustic pressure and particle motion thresholds in six sciaenid fishes. *J. Exp. Biol.* 211, 1504-1511. 10.1242/jeb.016196 18424685

[JEB243614C13] Ladich, F. (2019). Ears and hearing in vertebrates. In *Encyclopedia of Animal Behavior* (ed. J. E. Choe), pp. 46-53. Amsterdam: Elsevier, Academic Press.

[JEB243614C14] Ladich, F. and Fay, R. R. (2013). Auditory evoked potential audiometry in fish. *Rev. Fish Biol. Fish.* 23, 317-364. 10.1007/s11160-012-9297-z 26366046 PMC4560088

[JEB243614C15] Ladich, F. and Schulz-Mirbach, T. (2016). Diversity in fish auditory systems: one of the riddles of sensory biology. *Front. Ecol. Evol.* 4, 28. 10.3389/fevo.2016.00028

[JEB243614C16] Manley, G. A. and Clack, J. A. (2004). An outline of the evolution of vertebrate hearing organs. In *Evolution of the Vertebrate Auditory System* (ed. G. A. Manley, A. N. Popper and R. R. Fay), pp. 1-26. New York: Springer.

[JEB243614C17] Marone, F. and Stampanoni, M. (2012). Regridding reconstruction algorithm for real-time tomographic imaging. *J. Synchrotron Radiat.* 19, 1029-1037. 10.1107/S0909049512032864 23093766 PMC3480277

[JEB243614C18] Mokso, R., Schwyn, D. A., Walker, S. M., Doube, M., Wicklein, M., Müller, T., Stampanoni, M., Taylor, G. K. and Krapp, H. G. (2015). Four-dimensional in vivo X-ray microscopy with projection-guided gating. *Sci. Rep.* 5, 8727. 10.1038/srep08727 25762080 PMC4356984

[JEB243614C19] Mokso, R., Schlepütz, C. M., Theidel, G., Billich, H., Schmid, E., Celcer, T., Mikuljan, G., Sala, L., Marone, F. and Schlumpf, N. (2017). GigaFRoST: the gigabit fast readout system for tomography. *J. Synchrotron Radiat.* 24, 1250-1259. 10.1107/S1600577517013522 29091068 PMC5665295

[JEB243614C20] Myrberg, A. A., Jr and Spires, J. Y. (1980). Hearing in damselfishes: an analysis of signal detection among closely related species. *J. Comp. Physiol. A* 140, 135-144. 10.1007/BF00606305

[JEB243614C21] Paganin, D., Mayo, S. C., Gureyev, T. E., Miller, P. R. and Wilkins, S. W. (2002). Simultaneous phase and amplitude extraction from a single defocused image of a homogeneous object. *J. Microsc.* 206, 33-40. 10.1046/j.1365-2818.2002.01010.x 12000561

[JEB243614C22] Platt, C. and Popper, A. N. (1981). Fine structure and function of the ear. In *Hearing and Sound Communication in Fishes* (ed. W. N. Tavolga, A. N. Popper and R. R. Fay), pp. 3-38. New York: Springer.

[JEB243614C23] Popper, A. N. (1974). The response of the swim bladder of the goldfish (*Carassius auratus*) to acoustic stimuli. *J. Exp. Biol.* 60, 295-304. 10.1242/jeb.60.2.295 4832981

[JEB243614C24] Popper, A. N., Ramcharitar, J. and Campana, S. E. (2005). Why otoliths? Insights from inner ear physiology and fisheries biology. *Mar. Freshw. Res.* 56, 497-504. 10.1071/MF04267

[JEB243614C25] Rack, A., Garcia-Moreno, F., Schmitt, C., Betz, O., Cecilia, A., Ershov, A., Rack, T., Banhart, J. and Zabler, S. (2010). On the possibilities of hard X-ray imaging with high spatio-temporal resolution using polychromatic synchrotron radiation. *J. X-Ray Sci. Technol.* 18, 429-441. 10.3233/XST-2010-0273 21045279

[JEB243614C26] Ramcharitar, J. U., Higgs, D. M. and Popper, A. N. (2006). Audition in sciaenid fishes with different swim bladder-inner ear configurations. *J. Acoust. Soc. Am.* 119, 439-443. 10.1121/1.2139068 16454298

[JEB243614C27] Rogers, P. H. and Cox, M. (1988). Underwater sound as a biological stimulus. In *Sensory Biology of Aquatic Animals* (ed. J. Atema, R. R. Fay, A. N. Popper and W. N. Tavolga), 131-149. New York: Springer.

[JEB243614C28] Rogers, P. H., Popper, A. N., Hastings, M. C. and Saidel, W. M. (1988). Processing of acoustic signals in the auditory system of bony fish. *J. Acoust. Soc. Am.* 83, 338-349. 10.1121/1.396444 3343448

[JEB243614C29] Sand, O. and Michelsen, A. (1978). Vibration measurements of perch saccular otolith. *J. Comp. Physiol. A* 123, 85-89. 10.1007/BF00657346

[JEB243614C30] Schulz-Mirbach, T., Olbinado, M., Rack, A., Mittone, A., Bravin, A., Melzer, R. R., Ladich, F. and Heß, M. (2018). *In-situ* visualization of sound-induced otolith motion using hard X-ray phase contrast imaging. *Sci. Rep.* 8, 3121. 10.1038/s41598-018-21367-0 29449570 PMC5814409

[JEB243614C31] Schulz-Mirbach, T., Ladich, F., Plath, M. and Heß, M. (2019). Enigmatic ear stones: what we know about the functional role and evolution of fish otoliths. *Biol. Rev.* 94, 457-482. 10.1111/brv.12463 30239135

[JEB243614C32] Schulz-Mirbach, T., Ladich, F., Mittone, A., Olbinado, M., Bravin, A., Maiditsch, I. P., Melzer, R. R., Krysl, P. and Heß, M. (2020). Auditory chain reaction: effects of sound pressure and particle motion on auditory structures in fishes. *PLoS ONE* 15, e0230578. 10.1371/journal.pone.0230578 32218605 PMC7100961

[JEB243614C33] Von Frisch, K. (1938). Über die Bedeutung des Sacculus und der Lagena für den Gehörsinn der Fische. *Z. Vergl. Physiol.* 25, 703-747. 10.1007/BF00340903

[JEB243614C34] Walker, S. M., Schwyn, D. A., Mokso, R., Wicklein, M., Müller, T., Doube, M., Stampanoni, M., Krapp, H. G. and Taylor, G. K. (2014). In vivo time-resolved microtomography reveals the mechanics of the blowfly flight motor. *PLoS Biol.* 12, e1001823. 10.1371/journal.pbio.1001823 24667677 PMC3965381

[JEB243614C35] Witmer, L. M., Ridgely, R. C., Dufeau, D. L. and Semones, M. C. (2008). Using CT to peer into the past: 3D visualization of the brain and ear regions of birds, crocodiles, and nonavian dinosaurs. In *Anatomical Imaging* (ed. H. Endo and R. Frey), pp. 67-87. Springer.

[JEB243614C36] Wohlfahrt, T. A. (1932). Anatomische Untersuchungen über das Labyrinth der Elritze (*Phoxinus laevis* L.). *Z. Vergl. Physiol.* 17, 659-685. 10.1007/BF00339066

