# Supplementary material for: Revealing sound-induced motion patterns in fish hearing structures in 4D: a standing wave tube-like setup designed for high-resolution time-resolved tomography
Source: J Exp Biol. 2022 Jan 4;225(1):jeb243614. doi: 10.1242/jeb.243614 (PMC8778803; doi:10.1242/jeb.243614)
Supplement: Supplementary information [file jexbio-225-243614-s1.pdf]

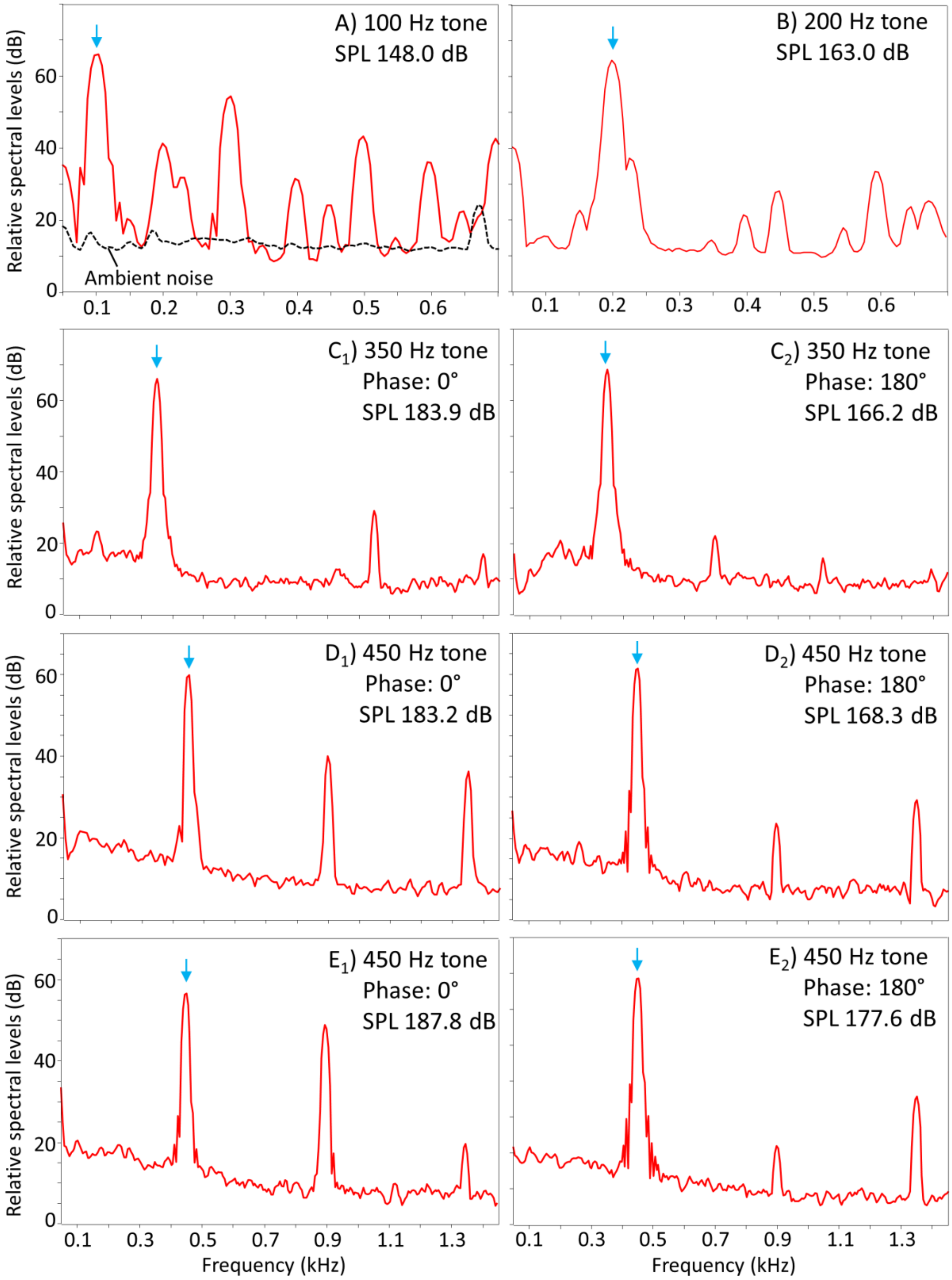


**Fig. S1. Ambient noise and tone stimulus spectra recorded in the one-shaker and two-shaker setups at the TOMCAT beamline at the Swiss Light Source. The red lines indicate the spectra of the recorded (A) 100 Hz, (B) 200 Hz stimuli, (C) 350 Hz, and (D) and (E) 450 Hz. ($C_1$, $D_1$, $E_1$) represent recorded spectra of stimuli when both inertial shakers were driven 0° in-phase whereas ($C_2$, $D_2$, $E_2$) depict spectra under the 180° out-of-phase condition. The black dashed line in (A) shows the spectrum of the ambient noise. Spectra were analyzed at a sampling rate of 44.1 kHz using a Hanning filter bandwidth of 10 Hz with 75% overlap. The blue arrow indicates the stimulus frequency.** SPL values indicated in each graph are given in dB re 1µPa.

Journal of Experimental Biology • Supplementary information

**Table S1. Sound pressure levels (SPLs) of the test frequencies and the ambient noise (AN) measured for the one-shaker and the two-shaker setups**. The studied samples are linked to the respective setup settings. Δ SPL [0°-180°], difference of measured SPLs when the two shakers were either driven 0° in-phase or 180° out-of-phase.

| **One-shaker setup** | **Frequency (Hz)** | **SPLs (dB re 1 µPa)** | **Δ SPL [0°-180°]** | **Species/ Sample** |
|---|---|---|---|---|
| **March 2019** | 100 (a) | 148.0 | | |
| | 100 (b) | 155.4 | | *C. auratus*/ isolated sagitta |
| | 200 | 163.0 | | |
| | *AN (hutch)* | *112.4* | | |
| **June 2019** | 100 (a) | 149.3 | | |
| | 100 (b) | 156.5 | | *D. rerio*/ whole fish |
| | 200 | 162.1 | | *D. rerio*/ whole fish |
| | *AN (hutch)* | *115.7* | | |
| **Two-shaker setup (standing wave tube-like setup), September 2020** | | | | |
| In-phase (0°) | 350 (a) | 183.9 | | *K. vitreolus*/ whole fish |
| Out-of-phase (180°) | 350 (a) | 166.2 | 17.7 | *K. vitreolus*/ whole fish |
| In-phase (0°) | 450 (a) | 183.2 | | *K. vitreolus*/ whole fish |
| Out-of-phase (180°) | 450 (a) | 168.3 | 14.9 | *K. vitreolus*/ whole fish |
| In-phase (0°) | 450 (b) | 187.7 | | |
| Out-of-phase (180°) | 450 (b) | 177.6 | 10.1 | |
| | *AN (hutch)* | *123.4* | | |

(a) & (b) represent two different amplifier settings (S.M.S.L. SA 36A Pro amplifier).

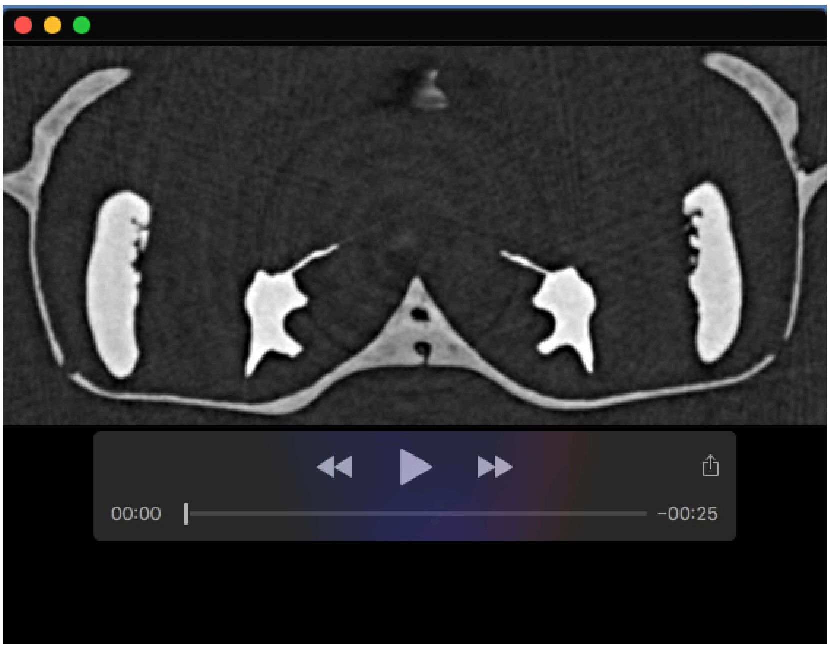


**Movie 1.** The saccular otoliths (shown in a transverse section) of *Kryptopterus vitreolus* (SL = 56 mm) display a symmetrical rotational movement when the fish was subjected to a 350 Hz pure tone stimulus under the 0° in-phase condition of the two-shakers setup. This sequence of tomographic images was generated by extracting the same slice from each of the 20 timing bins. Then the movie was generated looping the 20 images (saved as jpegs) and using a framerate of 20 fps.

Journal of Experimental Biology • Supplementary information
